# Glassy-like Transients in Semiconductor Nanomaterials

**DOI:** 10.3390/nano14050471

**Published:** 2024-03-05

**Authors:** Isaac Balberg

**Affiliations:** The Racah Institute of Physics, The Hebrew University, Jerusalem 9190401, Israel; balberg@mail.huji.ac.il

**Keywords:** glassy behavior, nano-semiconductors, transient currents, quantum confinement and Coulomb blockade, nonvolatile electrical and optical memories, quantum dots and MOSFETs

## Abstract

Glassy behavior is manifested by three time-dependent characteristics of a dynamic physical property. Such behaviors have been found in the electrical conductivity transients of various disordered systems, but the mechanisms that yield the glassy behavior are still under intensive debate. The focus of the present work is on the effect of the quantum confinement (QC) and the Coulomb blockade (CB) effects on the experimentally observed glassy-like behavior in semiconductor nanomaterials. Correspondingly, we studied the transient electrical currents in semiconductor systems that contain CdSe or Si nanosize crystallites, as a function of that size and the ambient temperature. In particular, in contrast to the more commonly studied post-excitation behavior in electronic glassy systems, we have also examined the current transients during the excitation. This has enabled us to show that the glassy behavior is a result of the nanosize nature of the studied systems and thus to conclude that the observed characteristics are sensitive to the above effects. Following this and the temperature dependence of the transients, we derived a more detailed macroscopic and microscopic understanding of the corresponding transport mechanisms and their glassy manifestations. We concluded that the observed electrical transients must be explained not only by the commonly suggested principle of the minimization of energy upon the approach to equilibrium, as in the mechanical (say, viscose) glass, but also by the principle of minimal energy dissipation by the electrical current which determines the percolation network of the electrical conductivity. We further suggest that the deep reason for the glassy-like behavior that is observed in the electrical transients of the nanomaterials studied is the close similarity between the localization range of electrons due to the Coulomb blockade and the caging range of the uncharged atomic-size particles in the classical mechanical glass. These considerations are expected to be useful for the understanding and planning of semiconductor nanodevices such as corresponding quantum dot memories and quantum well MOSFETs.

## 1. Introduction

The purpose of the work to be reported herein is to find whether there is a relation between the well-studied property of quantum confinement in semiconductor nanosystems and the transient electrical conductivity in them when the latter exhibits a glassy-like behavior. Noting that, commonly, two different audiences are interested in nano-semiconductor physics and glass physics, let us start by introducing the basic concepts of these two branches of condensed matter physics. The nano-quantum confinement (QC) effect is the consequence of the confinement of the electronic wave function to the size of a particle in the nanoscale [1,2,3,4,5]. Obviously, this effect becomes significant when the size of a system approaches that of an atom, thus yielding the widening of the electronic band gap in comparison with the one found in its macroscopic bulk [3,6]. The transition region between the atomic and macroscopic length scales is then of a few nanometers, and thus, the basic quantity that determines the electronic structure of the system is *d*, the diameter of the nanoparticles. In particular, the *d* value above which the system is practically bulk-like is known as the Bohr diameter. In the ensembles of nanocrystallites (NCs) studied here, the Bohr diameters are known to be 11.2 nm [7] in CdSe and 9.6 nm in Si [8]. The related increase in the optical band gaps through the transition are from the bulk value of 1.74 eV up to 3 eV for *d* = 2 nm in CdSe [1] and from 1.1 eV up to 1.9 eV for *d* = 3 nm in silicon [9]. These relations are commonly evaluated by optical [2], Raman [10], and scanning tunneling spectroscopy (STS) [11] methods. The other consequence of the small size of the NCs is the high electrostatic energy associated with their charging and its increase with the decrease in *d* [12,13]. In the case of nanoparticles (whether a NC, a metallic grain, or a metallic island), the tunneling between two of them requires an extra bias in order to overcome this energy, an effect that is well described by the double-barrier tunnel junction [14]. In an ensemble of nanoparticles, a charge carrier that charges a particle is essentially confined to (or trapped on) this particle, due to the Columbic energy involved in the charging of the next uncharged neighboring particles [15]. This electron localization is known as the Coulomb blockade (CB) effect [16]. Hence, the energy level structure associated with the local QC [3,9] and the local CB [17,18] determine the transport of the charge carriers throughout the macroscopic system. The sequential energy steps required to overcome the involved energy barriers on the route of the carrier are manifested experimentally by the current stairs in the corresponding current–voltage (I–V) characteristic. This manifestation is known as the Coulomb Staircase (CS) effect [17,19]. 

As generally common, we express the macroscopic electrical conductivity of a system as [20]

σ = *q*μ*n*, (1)

where *q* is the elementary electronic charge, μ is the resultant average mobility of the charge carriers, and *n* is their concentration. In the following, we generally assume that the charge carriers are electrons. Both μ and *n* can depend on T, the ambient temperature of the system. 

In the preset work, we studied σ(t), the time response of the conductivity to external time-dependent electrical or optical excitations, both during and after these excitations. Our study involved systems for which the QC, CB, and CS effects have been well demonstrated in many works by others [12,18,21] and by us [9,16,22]. Here, however, we focus on the changes in σ(t) with the variation in the basic parameter that determines these effects, i.e., the diameter of the nanoparticles, *d*. As far as we know, no such study has been reported thus far on any nano-semiconductors, and it has hardly been considered in any other nanosystems [23]. 

Probably the most known phenomenon associated with the transition from a non-equilibrium to an equilibrium state is glassy behavior [24,25,26,27]. Three principal transient features characterize this behavior [28]. First, a memory, which means that the properties of the system depend on the history of the past excitations [29]. Second, this history is recorded in a special way that is well known as *aging* [30,31]. This indicates that not only does a longer duration of the excitation, known also as the waiting time t_w_, yield a longer decay of the memory but also a quantitative scaling relation that describes it. Experimentally, this is exhibited by the observation that while the post-excitation decay of σ(t) is different for different t_w_s, the scaled σ(t/t_w_) is independent of t_w_ [32,33,34]. Here, we remark that while there are numerous systems that exhibit a memory of some kind, only those of them that exhibit this scaling feature can be considered as having a glassy-like behavior. Indeed, the glassy behavior is a great challenge to statistical mechanics and condensed matter physics, as well as being of great importance for practical applications [24,26,30]. The third feature that characterizes the glassy behavior is the “sluggishness” of the time dependence, i.e., a slower-than-exponential transient behavior. This may, or may not, be related to the above aging property and can be exhibited during both the excitation and the post-excitation decay processes. There are two basic possible contributions to this effect. The first is associated with the propagation of a particle (say, an atom in the glass or an electron in an ensemble) for which it is required to accumulate some thermal energy in order to overcome some barriers in the system, as during a hopping conduction [31,35,36]. The other possible contribution can come about due to a slow rearrangement of the environment [27,37] that may hinder the particle’s movements [38]. In particular, for the electrical systems, the “sluggishness” in manifested by a characteristic time constant τ of the rise and/or the decay of σ(t) which is much longer than any experimental macroscopic or intrinsic microscopic time of the system such as carrier scattering or recombination times [20] (which are less than a msec. in Si NCs [39,40]). 

Being interested in a quantitative description of the conductivity transients in general and the sluggish transients in particular, one represents such a transient by I(t), the experimentally measured current–time characteristic. Usually, the current rise and decay are determined by the linear rate equation d*n*/dt = g_n_ − *n*/τ, where g_n_ is the generation (or injection) rate of the mobile electrons and τ is the time constant for their disappearance [20,41]. Thus, during an optical or a bias excitation, the current rises as I(t) = I_e_(1 − e^−t/τ^), where I_e_ (**∝** g_n_) is the (steady-state) saturation current as t→∞. Correspondingly, upon the removal of the excitation, the relaxation or the decay of the current is given by I(t) = I_r_e^−t/τ^, where I_r_ is the initial current (which can be taken as t = 0 or t − t_w_) from which the decay relaxation is considered. Thus, the value of the characteristic time constant τ is empirically determined from the value of I at t = τ, i.e., from I(τ) = I_e_(e − 1)/e in the excitation and from I(τ) = I_r_/e in the relaxation [41]. When the excitation is superimposed on a background dc bias that provides a constant reference current I_st_, this current has to be subtracted from the above values of I_r_ and I_e_. Recalling that a sluggish I(t) is one that is slower than exponential, we also note that the most known analytical presentation of it is the stretched exponential function [42,43,44] in which (t/τ) is replaced by (t/τ)^β^ where β is known as the *decay exponent*. For the current decay, we have then that [44,45,46]

I(t)/I_r_ = exp[−(t/τ)^β^]. (2)

As clearly seen, under β < 1, the decay for t > τ is slower than that of the common exponential (i.e., that of the β = 1 case). When such a sluggish behavior is observed, β is usually assumed to represent a distribution of the values of the physical parameters that determine the measured value of τ. The smaller the β < 1 value, the wider the distribution [44]. In glasses, this can be the distribution of local viscosities [25], while in nanocomposites, this can be the distribution of the tunneling barrier widths between nearest neighbors or other random potential fluctuations through the system [47,48]. In particular, the latter distributions can be associated with grains’ charging [49,50] and/or level quantization of neighboring nanocrystallites [1,40]. On the other hand, in some nanosystems, the data fit the logarithmic relation [33,51]

ln[I(t)/I_r_] = A − Bln(t), (3)

where A and B are fitting parameters [43] that enclose more detailed physical information of the system Here, we remark that in glasses the kinetics can vary from stretched exponential [24] through ln(t)-like to 1/t-like dependencies [52,53,54,55,56]. Correspondingly, we define here a system as glassy-like if all the above-mentioned three “glassy” features are exhibited by its characteristics, and we refer to such systems as having a simple glassy behavior (SGB). 

However, the observation of SGB as such cannot provide the specific information as to the mechanisms that lead to this behavior, and thus, in order to unravel these mechanisms, additional detailed and/or specific studies are needed. In the case of electrical systems, this information can be provided by studying the dependence of I(t) on experimentally controllable parameters such as the temperature T, the applied bias V, and the concentration *n* [43,57], as well as the configuration of the measurement (e.g., field-effect-like [23,54]). In the case where the excitation of the conductivity is by illumination, this also includes the illumination intensity, F, and the photons’ energy [48,58,59]. Concentrating here on the SGB in systems with electrical interactions, we define these systems as having a Coulomb glass behavior (CGB). This is in order to distinguish this rather broad family of electronic systems [28,38,60,61,62,63,64] from the specific widely studied system of the electron *glass* [34,57,65,66,67,68], which is just one member of this family. This member, the so-called “intrinsic” electron glass system [69], is unique by being determined by the Anderson disorder localization at a high *n*. Here, we concentrate on nanosystems that exhibit the QC-CB effects for which we examine the possibility of a CGB [70]. This is by the measurements of the transient I(t) under various conditions, with the aim to resolve three basic questions. The first is whether the SGB is indeed exhibited by the I(t) characteristics in these semiconductor nanosystems. The second is, if so, how do the QC and CB effects influence the glassy characteristics? The third and more fundamental question is whether an observed SGB in these systems is just an artificial empirical–experiential presentation of experimental results or is it due to some basic physical resemblance between the collective Coulomb mechanism [70,71] and the collective mechanical (say, the viscosity) mechanism that results in SGB in the classical glass [24,35,67]. In the work reported here, we tried to respond to these three questions with an experimental study on two types of nano-semiconductors for which the QC-CB-CS effects have been previously observed by others [1,5,6,63] and by us [3,40,42]. 

To set the stage for our specific study of the possible connection between the phenomena of glassy behavior and the nano-nature of the QC-and-CB effects, we start with a brief review of two studies in which nanoparticles were involved and the transient conductivity in the lateral configuration was measured. The first study was carried out on porous silicon [60] in which Si NCs were embedded in an amorphous Si-O-H matrix as peas in a peapod [72,73]. The origin of the convincingly obtained SGB in this system was suggested in [33] to be of an atomic–ionic nature (sometimes referred to as an extrinsic glassy mechanism [69]), but the QC and related effects were considered there. On the other hand, in the many other works on porous silicon where the QC and related effects have been well established [6,73], there was no mention of the glassy behavior. The other study of I(t) that convincingly demonstrated a SGB in the lateral conductivity configuration was carried out on a discontinuous metal film that consisted of isolated nanosize non-spherical metallic islands [61,74]. However, being metallic, the CB effect was considered, but of course, the semiconductor-like QC effect is not to be expected. In particular, we remark that in both [60,61], no *d*-dependent was considered, and no temperature dependence of the conductivity transients was reported.

Following the above, in this work, we studied the transient conductivity in systems for which we have extensively reported the QC and CB effects [3,75,76,77] but for which, as far as we know, no glassy behavior has been demonstrated. Thus, the possible relation between the three features of the SGB and the firmly confirmed QC in them is thus far unknown. Correspondingly, we report here results of the measured I(t) as a function of the size of the nanocrystallites, *d*, and the ambient temperature, T. For simplicity and clarity, we concentrate in this paper on results that we obtained by applying the very simple two (or four)-probe measurement in the lateral film configuration [34,60,61,70,78]. As seen below, this enables us to establish and explain the relation between the SGB and the QC-CB phenomena in semiconductor nanosystems. The samples we studied are nano-CdSe photoconductors [1,76,77] and the nano-Si-SiO_2_ composites [3,22,75] on which we conducted optical [3,9], electrical [15,76], and nanospectroscopy [11,77] measurements. In [77], we detailed our results on nano-CdSe, and in [3,9,40,76,79], we detailed our results on nano-Si-SiO_2_. Here, we mention that such systems have already been shown to result in quite a few optoelectronic applications [5,13], the conspicuous of which are quantum dots [50,63,80,81,82,83] and quantum well [47,48,58,84,85] devices. We suggest then that utilizing the glassy behavior for the analysis and guidance of existing and novel optoelectronic nonvolatile memories appears to be quite promising.

The content of this paper is as follows. In Section 2, we provide brief descriptions of the samples that we studied and the I(t) measurements that we carried out on them. In Section 3, we present our results on the transient photocurrent in the studied nano-CdSe samples, where the emphasis is on the sluggishness of the I(t) dependence, and we discuss related results in the literature. In Section 4, we present the results of the bias-induced transient current I(t) in our nano-Si-SiO_2_ composites for different concentrations and sizes of the Si nanocrystallites. This is under different bias and temperature conditions for both the excitation and relaxation regimes of the I(t)s. We emphasize there the aging effect and make a comparison with relevant observations in the literature. In Section 5, we summarize the main results and consequences that emerge from our study and the above comparisons, thus proposing the relation between the SGB and the QC-CB effects in the studied systems and beyond. In particular, we point out the basic physical principles that determine the I(t)s and the similarities that lead to the glassy behavior of the presently studied and other nanosystems. In Section 6, we conclude by mentioning the main advancements that we made in the present work.

## 2. Materials and Methods

Our CdSe films, which were grown with a chemical bath deposition on quartz substrates, were 800–1200 nm thick. The size of the crystallites that form in the film is determined by the temperature at the deposition, T_d_, such that *d* = 7.5 + 0.07 T_d_ increases from 8 to 13 nm when T_d_ varies between 0 and 80 C. The QC controlled band gap was found with light absorption measurements to vary from 2.0 eV to 1.84 eV [76]. The *d* values were determined using Raman spectroscopy [72] SEM, HRTEM, and STS [42,77,78,80,81] measurements. We also found that the activation energy of the coplanar dark conductivity increases from 0 to 0.55 eV as the size of the CdSe nanocrystallites decreases from 11 nm to 7.5 nm [77]. Considering the widely studied photoconductivity in such nano-CdSe systems [1,70,82], and in order to minimize the problems associated with carrier injection through the contacts [82], we used the advantage of spatially uniform illumination excitation throughout the sample by measuring the photocurrent in the coplanar 2-probe or 4-probe configurations. The electrical contacts to a given sample consisted of pairs of evaporated Al, In, or Ag strips. The I(t) responses to rectangular-like illumination pulses [84,85] that were provided by lasers of various wavelengths were measured by applying a constant small dc bias. The basic features of the observed I(t)s were found to be independent of the contact types and the wavelength of the illumination.

**Figure 1:**
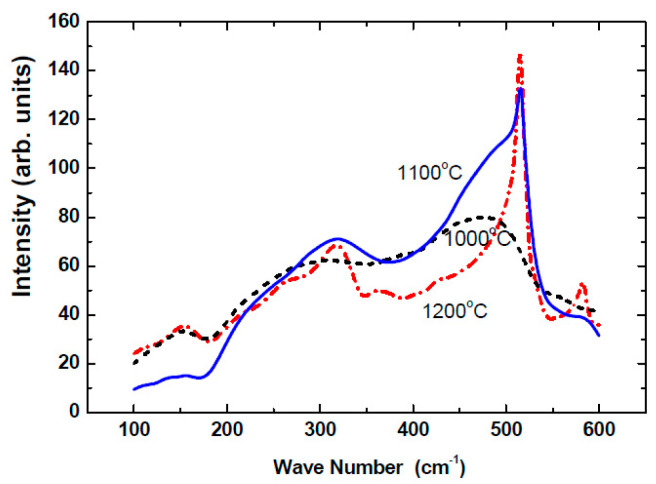
The Raman spectra as obtained on samples of the same intermediate x value for three slide samples that were annealed at different temperatures. Note the diminishing amorphous Si hump (around 480 cm^−1^) and the emerging of the crystalline Si peak (around 521 cm^−1^) with the increase in the annealing temperatures. For more details of our Raman measurements see [10].

As detailed previously [22,75,86], our nano-Si samples were deposited with the co-sputtering of two source materials (targets): high-purity sintered Si pallets and pure fused quartz. The sputtering process was carried out using a plasma of Ar ions that bombarded the targets with energies that were enough to eject clusters of a few atoms from the targets and deposit them on elongated, 13 cm long and 1 cm wide, quartz slides as substrates [75,86]. Our 3 mm wide and 600–1200 nm thick films were such that, during the sputtering process, one end of the substrate was adjacent to the Si target and the other to the fused quartz target. The deposition conditions that we applied yielded a mixture of Si and SiO_2_ phases. Various examinations of the resulting composites indicated that, at the slide end adjacent to the Si target, the fractional volume content of the silicon phase in the films, x, was about 90 Si vol.%, while at the other end, it was about 10 Si vol.%. Since the x values are very frequently mentioned in the paper, we will occasionally refer to them throughout the paper just as x without adding the Si vol.% units. To ensure the formation of Si NCs in the films, the slide samples were annealed at 1100–1200 °C in a N_2_ atmosphere for about an hour. In particular, for an annealing temperature T_a_ ≥ 1150 °C, the Si phase of the films practically consists only of NCs [16,75,86,87]. This conclusion was derived from our Raman [10,16] and HRTEM [75,86] measurements. For example, as shown by our Raman spectra in Figure 1, the crystalline Si peak (at 521 cm^−1^) “overcomes” the amorphous Si hump (around 480 cm^−1^) when T_a_ was in the 1100–1200 °C range, thus indicating that the entire Si phase consists of NCs. This is in accord with many previous studies on the formation of Si NCs, where different fabrications of Si-SiO_2_ films were applied [85,88,89]. In particular, it will be of importance below that in [85] it was concluded that for “T_a_ > 1000 °C most of the Si NCs are electrically isolated from each other by the surrounding silicon oxide matrix and that the photoexcited carriers will be mainly localized to the respective Si nanostructures”. Following the above, we call our entire (13 cm long) film after the annealing the “slide sample” and a (2 mm wide) specific sector of it, which is defined by its x value, a “sample”.

**Figure 2:**
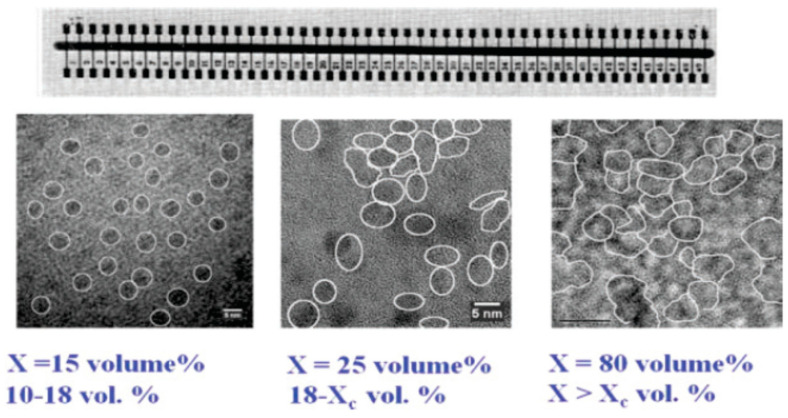
The deposited contact configuration on our slide sample and the HRTEM images as obtained on three samples of a slide sample after annealing at T_a_ = 1150 °C. The three images are typical for the range of very small x values, the range of intermediate x values just below the geometrical percolation threshold x_c_, and the range of large x values above this threshold. As given by Equation (4), the average size of particles, from left to right, is 4 nm, 5 nm and 8 nm. For more details, of our samples see [22,75,86,87].

As shown in Figure 2, for the electrical conductivity measurements and for the definition of the x sectors, we have deposited thin (Al, In, or Ag) contact-finger electrodes perpendicular to the slide-film [75,86]. No significant differences in the resulting I(t) behaviors were found with the use of different metal electrodes. To establish that the above Raman findings are associated with the Si NCs and not with a continuous Si phase, we obtained HRTEM images for a slide sample that was annealed at 1150 C [75,86]. The variation in the size and concentration of the NCs with x is exhibited in Figure 2. In this figure, one can also see that the possible current routes in the samples take place in between isolated NCs in the low-x samples and through touching NCs in the high-x ones. The geometrical percolation threshold of touching NCs and the sharp conductivity rise that accompanies it, x_c_, were found to be around x = 35 Si vol.% [16,75,87]. As seen in the figure, there is a monotonic increase in *d* with x. The empirical relation we found between the average diameter of the NCs, *d*, and the x value of a sample is roughly given by [9]

*d* ≈ *d*_0_(x − x_0_)^1/3^, (4)

where the exact *d*_0_ and x_0_ values vary somewhat with the conditions of the samples’ fabrication. Typically, however, in our samples, *d*_0_ ≈ 2 nm and x_0_ ≈ 8 Si vol.%, yielding a variation in *d* from 3 nm to 10 nm along the 10 < x < 90 Si vol.% film of the slide sample. For all the Si-SiO_2_ slide samples studied here, we have confirmed the presence of QC with optical absorption [3,75,79,90], transport [22,75,90,91], charge [79], and local structure spectroscopies [11,86,90]. In particular, the former showed a 1.4 to 1.8 eV shift for 3 < d < 8 nm.

In the study of our above nano-Si-SiO_2_ composites, we followed the transients of the conductivity during and past the application of a rectangular pulse of bias or illumination. In the measurements, we applied the basic common protocol for I(t) measurements, which consists of a small “background” dc voltage on which the bias V [33,60,61,92], or the illumination intensity F [58,80,84], have been superimposed during the excitation time t_w_. The constant background bias is required for monitoring the current after the termination bias and throughout the entire photocurrent measurement. 

The I(t) measurements on various x and *d* samples were carried out as a function of V and/or F and the ambient temperature T. Considering the advantages associated with stationary point-by-point readings [93], we used static measurement instruments to measure both the I–V and I(t) dependencies. This was enabled by the fact that the immediate rise and drop times of the observed current response (such as the resultant RC time of the circuit or intrinsic carrier lifetimes in the system) were considerably shorter than the I(t) features of interest, for both t < t_w_ and t > t_w_. In general, for both types of excitation pulses, the current increased towards some saturation value I_e_ [41,94], and after pulse termination, there was always a quick drop in the current that was followed by a very slow decay of I(t) towards I_st_ the dc current due to the background bias. In the case of excitation by illumination, this behavior is the well-known persistent photocurrent (PPC) [44,47,48,58,59,95,96,97] phenomenon, and under bias excitation, it is known as the memory [51,60,64] or retention [49,63,65,93] effect. Correspondingly, the focus in this work is on the I(t) − I_st_ characteristics.

## 3. I(t) Results on Nanocrystalline CdSe under Photoexcitation

**Figure 3:**
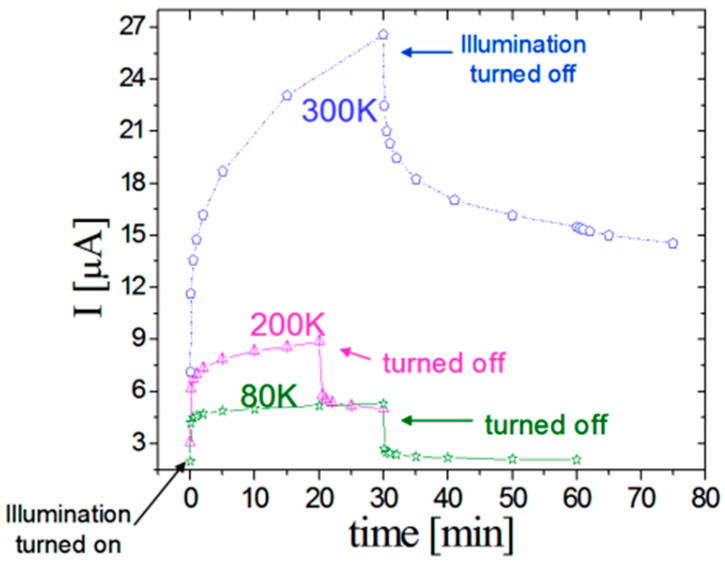
Photocurrent–time characteristics under three ambient temperatures as measured on a film of CdSe NCs with an average diameter *d* = 11.6 nm. The separation between the two identical macroscopic electrodes of a sample was 2 mm, and the applied bias was 1 V. The light source was a 530 nm diode laser that was turned on and off, as indicated in the figure. More details of these samples and their properties can be found in [42,76,77].

In this section, we show that the transient photoconductivity in ensembles of CdSe NCs exhibits the basic features of the glassy behavior. In the measurement, an application of a constant reference bias of 1 V yielded a corresponding background dark current I_st_. For the excitation of much higher currents, a strongly absorbed light from a diode laser illuminated the sample. In Figure 3, we show typical current–time characteristics that were obtained by the application of an on–off spatially uniform illumination [58,87,97,98,99] between the two coplanar contacts of a sample.

As seen in Figure 3, the observed characteristics consist of two regimes. The excitation, illumination-on, regime (here the 0 < t < 30 min. time interval) and the decay relaxation, illumination-off, regime (here the t > 30 or t > 20 min. interval). During the excitation, I(t) has a concave shape, while during the decay relaxation, it becomes convex. This basic behavior of the I(t)s, which is quite typical of photoconductors [41,99,100], was previously observed in many nano- [42,58,72,85,99] and non-nanosystems [43,44,84,101,102,103]. Usually, the corresponding long relaxation decays are described by a stretched exponential, as given in Equation (2) [44]. These were attributed to trapping–detrapping processes with special types and/or concentrations of defect states [43,87,104,105,106]. Here, we note that the basic I(t) features exhibited in the figure have also been observed following a bias pulse on other nanosystems of CdSe [63,82,107] as well as on nanosystems that are based on metallic particles [51,61,74] and various quantum semiconductor structures [60,92,108,109]. Before we turn to the microscopic mechanisms that may account for the characteristics shown in Figure 3 (and our results in [77]), we recall that macroscopically there is a basic physical difference between the excitation regime and the decay relaxation regime. While in the former the I(t) is dominated by the approach to a steady state (which results from the balancing of the carrier’s generation or injection by their recombination), in the latter, the dominant mechanism that determines I(t) is the decay of the system towards its equilibrium state. Here, we remind that such decays in both photoconductors [43,44,51] and glasses [24,32,102] were described in the literature by a stretched exponential, as in Equation (2), logarithmic decay, as in Equation (4) [33,56], or by power-law-like decay, as in [29]. 

Turning to explain the results in Figure 3, we consider two different microscopic mechanisms that have been proposed previously for nano-CdSe systems. In [82], it was actually suggested that the NCs play the same role as stationary defects in a semiconductor, where there are *n* “free” carriers and *n_t_* charged defects. Here, we add that a possible more extended version of this explanation suggests that with the increase in t during the excitation, both *n* and *n_t_* increase, but the increase in *n_t_* causes the decrease in the effective mobility of the electrons due the electrostatic repulsion. Then, following the termination of the excitation, there is a release of the trapped electrons, which provides the persistence of the current. The much longer decay relaxation time than the expected detrapping time can be attributed a priori to be due to an activated hopping between the NCs. In fact, while the authors of [82] mentioned spin glasses, they still interpreted their data in terms of the defect models [20,43,44,94,101], concluding that these are “more closely related” to their nano-CdSe system. A “more glassy”-like [110,111] interpretation was proposed, however, in the pioneering work of [70] in which, following the excitation of the photoconductivity and the dark conductivity [112], it was concluded that, as in metallic disordered arrays, Coulomb-induced correlations “play a major role in the transport through CdSe NC solids”. In other words, unlike the interplay between *n* and *n_t_* in [82], they suggested, however without specifying how, that it is the rearrangement of the “charged” NCs that is responsible for the observed long relaxation times of the photoconductivity in their nano-CdSe system. Here, we propose a more specific meaning to the collective or hierarchic effects that were mentioned in [70] by adapting the simple model of [61] that suggests that the decay relaxation towards equilibrium is significantly extended due to the response of the hopping electrons to the varying charged landscape that was established during the excitation. The corresponding decay time then is much longer than the conventional decay time of hopping processes [113] in general and in local transport processes in nano-CdSe [77] in particular. 

Considering the quantitative details of the results shown in Figure 3, we found that the values of τ and β, which we derived by fitting the observed decays of I(t) to Equation (2) [42], are independent of the illumination wavelength (between 655 and 488 nm) and (within a factor of 10) of the illumination intensity. On the other hand, the values of τ and β were found to depend on the crystallites’ diameter *d* and the temperature T. As seen in Equation (2), the value of τ during the decay relaxation is simply the value of t at which I(t = τ) = I_0_/e. Considering the temperature dependence in Figure 3, we note that usually [41,47,59,66,94] the increase in temperature promotes the decrease in τ (say, due to an enhanced electron–hole recombination). Such a temperature-activated decay time behavior is described by

τ = τ_0_exp(E/kT), (5)

where τ_0_ is the relevant system-dependent quantity, E is the energy barrier involved in the decay process, and kT is the thermal energy. In our nano-CdSe system, we found however the opposite behavior [42]. This is that τ increased from 6 min at 80 K to 100 min at 300 K. Contrary to the above-mentioned more common behavior of Equation (5), such a dependence or even both possible trends of the τ(T) dependence have been found in quite a few systems that exhibit a persistent photocurrent (PPC) [34,43,44,58,59,101,114,115,116]. In particular, we remark that exactly the same dependence as ours [42] has been found in polycrystalline Cu_2_ZnSnS_4_ [105], and there it was interpreted as due to hopping conduction. Of special interest here is that in quantum wells or quantum dot systems in which PPC has been observed, τ was found to be activated as in Equation (5) at higher temperatures, while at lower temperatures, it was found to saturate [48] or even decrease with decreasing temperature [58,59,84,99], as we found in the entire T range that we studied here. The results in those quantum systems were interpreted to be due to potential fluctuations that may [58], or may not [59], be applicable to our nano-CdSe system. On the other hand, the fact that, as in our nano-CdSe system, those systems exhibit the QC-CB effect suggests that these effects also play a dominant role in the τ(T) dependence that we found here. Interesting here is the fact that the peaked τ(T) behavior found in a-Se [102] was attributed to particular defects and to a “charge adjustment” that is induced by a “slow reorganization” of the trapped charges. Moreover, it has also been found in [102] that the photocurrent decay rate decreases with the increase in the illumination pulse duration. The importance of this observation in the present context of the glassy behavior and the photoconductivity in nano-CdSe is that, while not stated explicitly by the authors, they actually connected the rearrangement of the trapped charges with the property of aging. 

**Figure 4:**
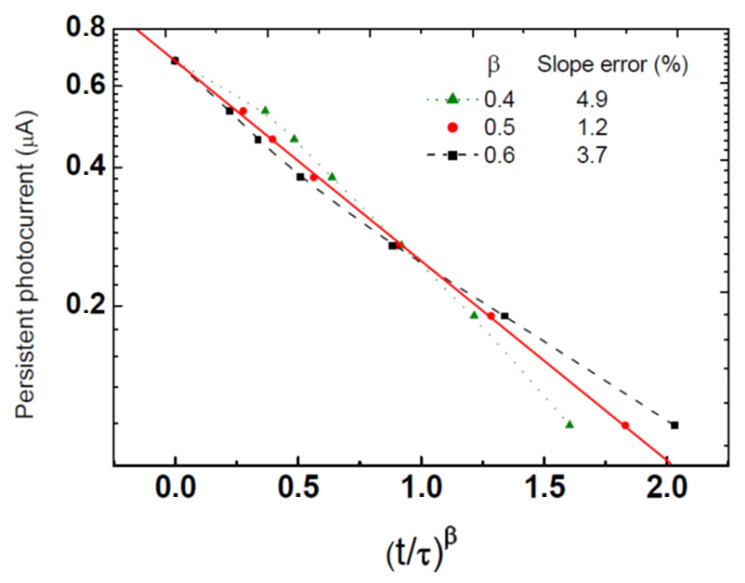
The derivation of the stretching exponent of the photocurrent for a sample of *d* = 11.2 at T = 80 K. The curves shown are the least squares fittings to the stretched exponential function of Equation (2). The “slope error” here represents the quality of the fit for possible β values [42].

Following that the interpretations of the T dependence of I(t) were generally derived in the literature on the basis of both the τ and β, as defined by Equation (2), we also determined the temperature dependence of the “stretching exponent” β. This was achieved by finding the best-fitted value from the slope of logI(t) as a function (t/τ)^β^ using the already known τ. For example, as shown in Figure 4, the best β value at T = 80 K is β = 0.5. 

For the T regime that is examined in Figure 3, we found that β decreased with increasing temperature [42]. In comparison, we note that τ(T) and β(T) results such as ours have been obtained on quantum wells [47,58,99] and photoconducting alloys [59,84,117] and were interpreted then in terms of local potential fluctuations, while for various continuous [43,101], polycrystalline [59], and amorphous silicon [46], such results were attributed to particular defects. Here, we mention that Dixit et al. [43] found that the increase in the dark conductivity and/or the defect concentration yielded a variation in the photocurrent I(t) from that of Equation (2) to that of Equation (3). As confirmed in [51], the parameters A and B, just as the parameters τ and β here, may be temperature-dependent. Following the long-called-for understanding of the combination of the above-mentioned processes in nano-semiconductors [63,78], we will elaborate in Section 5 below on them and the corresponding temperature dependencies by using first-principles physics within the framework of the glassy behavior. 

Turning to our interest in the possible influence of the QC and related effects [16] on the glassy behavior [25] of nano-semiconductors, we have studied the dependencies of τ and β on *d*. As far as we know, such *d* dependencies have not been studied on any system that exhibited these effects. Finding that τ(d) increases with increasing d while β(d) decreases with it [42], we conclude that the observed I(t) relaxations are affected by the influence of the QC, CB, and CS effects via their impact on the electrostatic energies involved in the electronic structure [1,3,21,84] as well as on the transport processes in that nanosystem [13,118]. In particular, trapping–detrapping processes are carried out here by a tunneling to neighboring NCs mechanism, such as already observed in nanosystems of Si [17,18] and CdSe [63]. This is of course the essence of the retention property of electrical and/or optical nonvolatile memories in nano-semiconductor devices [108,109,118,119] where the trapping is associated with bulk or surface quantum-confined states [1,5] rather than with chemical or structural defects [120,121]. In fact, as others [63,122], we found [79] that under saturation of injection due to bias application, the concentration of embedded charges in these systems does not surpass the concentration of the nanocrystallites (of the order of 10^18^ cm^−3^), which means that, on average, there is less than a single retained electron per NC [16,63,79,122]. The increase in τ with *d* that we found here can be attributed to the smaller repulsive force of the corresponding Coulomb blockade [13,16,17,123] and thus to the longer range that the electrons can travel before they are captured (or trapped). One may also argue that the decrease in β with the increase in *d* is associated with the nano-nature of the system due to the broader distribution of the QC levels and/or tunneling barriers associated with their variation between different NCs. 

Let us recall now that the major feature that distinguishes a glassy behavior from other types of memories is the aging effect, i.e., the scaled increase in the observed τ with the increase in the excitation “waiting” time t_w_. We suggest here that this also applies to our nano-CdSe under the illumination excitation as follows. While not presenting this scaling of the aging, the basic qualitative property of the effect (i.e., the increase in τ with the increase in t_w_) has been reported for glassy a-Se [102] under illumination excitation and, of particular interest here, for nano-CdSe [107] under bias or illumination excitation. To support our suggestion, we also recall that, as mentioned above, the scaling behavior of I(t) was well demonstrated in porous silicon under bias excitation [33,60] which as in our nano-CdSe [77] the transport in porous silicon [72] is determined by semiconductor NCs. For a further support to this suggestion, we compare the memory manifestation due to bias excitation in this porous silicon and in our photoconducting nano-CdSe. 

**Figure 5:**
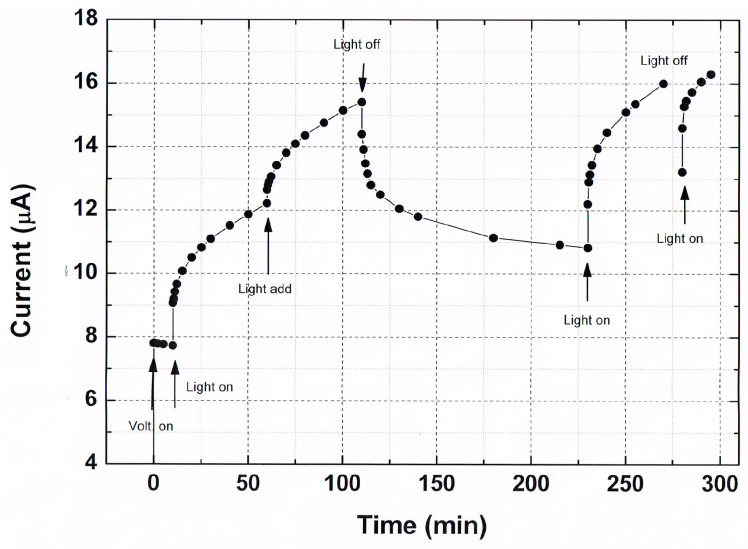
The I(t) response under a subsequent application of illumination pulses. The measurement was carried out on a similar sample (but here for *d* = 11.5 nm) and in similar conditions to those used for the derivation of Figure 3. Note that the memory exists both during the excitation and during the decay relaxation regime of I(t).

To carry out this comparison, we used the common protocol of on–off illumination [58,84], finding, as shown in Figure 5, that the measured I(t) of each subsequent illumination pulse is added to that of the previous pulse both during its excitation and after its termination. This superposition is very similar to the superposition dependence of the current under bias excitation that was observed by Borini et al. [60] on porous silicon. Adding to this, the memory and sluggishness of I(t), as seen in Figure 3 and Figure 4, and the mentioned aging effect in [60,107] strongly support the conclusion that our above results represent the three features of glassy behavior. To this, we add here that our above-mentioned *d* dependence of I(t) indicates that the glassy behavior in both systems is coupled to the QC and CB effects.

**Figure 6:**
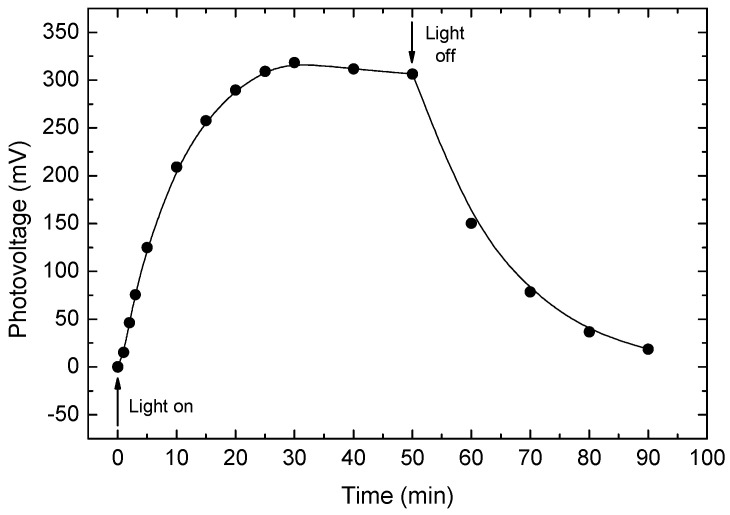
The time dependence of the lateral photovoltage as measured on a non-annealed Si-SiO_2_ sample in a coplanar configuration under open-circuit conditions. A He-Ne laser provided the illumination. The photovoltage is generated here by the gradient of *d* and x along the sample, as described in [81]. The special observation to be noted is the initial slow decrease in the current even before the termination of the illumination.

To validate the generality of the above transient photoconductivity characteristics in systems that exhibit the QC and CB effect, we show in Figure 6 the transient of the lateral open-circuit photovoltage between two subsequent contacts, which we obtained on our Si-SiO_2_ nanocomposites. In our samples, the inhomogeneity of the sample that is required for that photovoltage is provided by the gradients of *d* and the conductivity that accompany the change in x above x_c_ along the slide sample, as we detailed in [81]. Here, we have chosen to present the behavior of I(t) via our measured lateral photovoltaic effect (LPE) in order to demonstrate the usefulness of studies similar to ours for optoelectronic applications such as the well-known Global Positioning System (GPS). As in general [20,94] and as we confirmed here, this transient of the photovoltage is related logarithmically to the transient photocurrent. Indeed, as shown in Figure 6, the behavior we found is very similar to the behavior that we obtained in Figure 3 and Figure 5 for our ensembles of CdSe NCs. Here, however, we also see a decline in the photocurrent while the illumination excitation is still on. Such I(t) characteristics have been previously studied in detail as a function of the postdeposition annealing temperature T_a_ in a differently prepared Si-SiO_2_ nanocomposite [85]. The interesting finding there was that the initial decline during the photoexcitation was enhanced with the increase in T_a_, i.e., with the formation of the NCs. 

It is to be mentioned that while in [85] this behavior was found only for samples that were annealed above T_a_ = 650 °C, we found it already for our non-annealed samples. We attribute this to the well-known difference between the two types of samples [88,89], i.e., by the fact that unlike the deposition by electron beam evaporation as in [85], in our co-sputtered samples, the Si clusters are provided already during the deposition. This makes our non-annealed Si-SiO_2_ nanosamples similar to porous silicon [72] for which, as mentioned above, the glassy behavior was well confirmed [33,60]. Support to our interpretation of the result in Figure 6 in terms of the glassy behavior will be given in the next section by the I(t)s that we obtained with a bias excitation of our annealed Si-SiO_2_ nanocomposites and by comparison of those I(t)s with results that were reported in systems of metallic nanoparticles [61] that clearly demonstrated the glassy behavior. 

Here, we have to add that while a peaked I(t) behavior of the photoconductivity is quite typical to photoconductors and is usually interpreted to be due to a recombination–trapping process in particular defects [101,124,125], we suggest here that the mechanism that dominates our observed I(t)s is associated with the common nano-nature of our CdSe and Si-SiO_2_ systems. Our suggestion is supported by the above-described increase in τ and the decrease in β with the increase in *d*. This is since small changes in *d* cannot be expected to yield a variation in the known defects in bulk semiconductors, but such changes in *d* around the Bohr diameter [1,7,8] can vary the nano-QC and CB effects in both nano-CdSe [1] and in our nano-Si [3,79]. A strong support for this argument is provided by the results of [85], where the time dependence of the I(t)s was found to vary with the energy of the optical absorption edge of the Si-SiO_2_ composites. This result does not only show that the I(t) characteristics change with the value *d* but they directly prove that the characteristics vary with the band-induced shift due to the QC effect. In the next section, we show that the glassy behavior in these composites also has to do with the nano-nature of the system, rather than with semiconductor defects. 

## 4. I(t) Results on Si-SiO_2_ Nanocomposites under Bias Excitation

**Figure 7:**
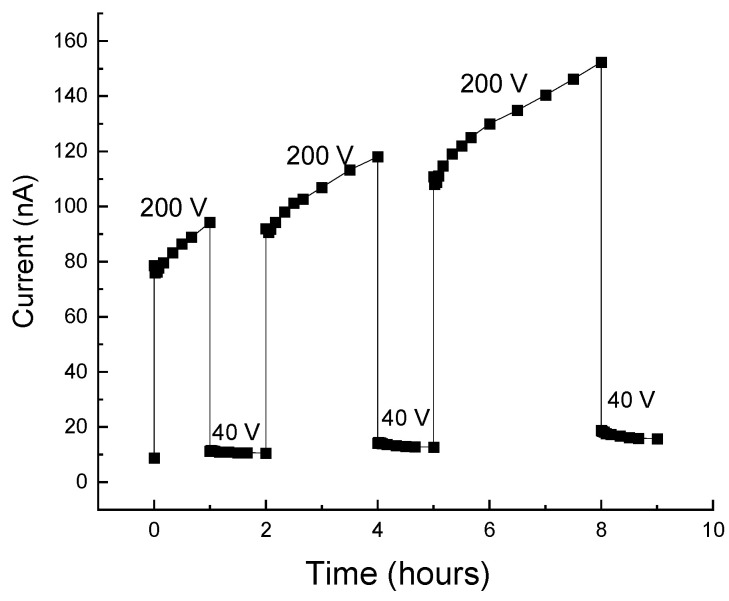
An I(t) response characteristic of a bias application of three subsequent rectangular pulses to a sample of x = 75.3 Si vol.% that was annealed at 1150 °C. The center of the distribution of the embedded NCs in this sample is *d* = 8 nm. Note the concave shape of I(t) as in the bias excitation in both Figure 5 and Figure 6. In this room-temperature case, the V = 40 V background was the minimal V value needed for monitoring the current decay past the excitation. The 200 V bias was chosen to overcome the initial current decline and contact effects that will be discussed below.

In our study of the Si-SiO_2_ nanocomposites, the transient conductivity was usually excited by an applied bias. Here, we start by showing that the three features of glassy behavior are exhibited by our corresponding I(t) characteristics. However, within the scope of the present paper, we will not be concerned with the particular value (or amplitude) of the current, which depends on the finer details of the deposition and annealing procedures during the sample preparation [3,16,22,75,79,86]. Rather, we will concentrate on the basic qualitative features of the I(t) characteristics. In particular, as in other relevant works [33], we found that the use of different samples during the study of the I(t)s does not seem to affect any of the conclusions and interpretations that will be derived further below. In Figure 7, we show the I(t) characteristic of a sample of x = 75.3 Si vol.%, for which the Gaussian distribution of the NCs’ diameters [9] is centered at *d* = 8 nm. In the corresponding measurement, a 40 V dc bias determined I_st_, the background dc current, which is assumed to be close to the equilibrium (Ohmic-dark) conditions. Upon the application of a rectangular pulse bias of 200 V according to the common protocol of such bias applications [33,51,60,61,62,92], the sample was driven to the non-equilibrium regime. In the figure, we show the I(t) response to the application of three consequent pulses with durations or (waiting or excitation) times t_w_ of 1, 2, and 3 h. In view of our interest in the glassy behavior [27,35,36], the focus of our study is on the concave I(t) at the excitation regimes and the convex I(t) at the decay relaxation regimes past the corresponding excitations. One the other hand, the experimental very sharp initial rise in the current at the onset of the pulse and very sharp drop in it at the pulse termination are of no interest in the present work.

Now we note that these qualitative features are very similar to those observed in many systems during bias excitations [33,51,62,92] or photoexcitations [41,58,59,84,85,90,126], as in the above Figure 5. 

The glassy feature of memory is clearly exhibited in the figure by the fact that the I(t) of each subsequent pulse picks up where the I(t) of the previous pulse left off. The times involved here (hours) are much longer than any microscopic (recombination-like [20]) or macroscopic (circuit-RC-like [40]) time constants of the sample (less than seconds), thus confirming the sluggishness of the relevant transport process during and past the pulse application. Having these memory and sluggishness features of the glassy behavior, we have checked the fulfillment of the most characteristic feature of the glassy behavior, the aging. Hence, applying the standard procedure for its confirmation [33,60,61], we measured the I(t) decay that followed the termination of the bias pulses. This was by monitoring the current decay ΔI(t > t_w_) = I(t > t_w_) − I_st_, where I_st_, = I(t = 0) is the current due the background bias of 40 V, which enabled us to follow the current after the termination of the excitation pulse. The aging effect is revealed then by presenting the dependence of the normalized current decay ΔI(t)/(I_0_ − I_st_)) on the normalized time (t-t_0_)/t_w_, where I_0_ is the current at t_0_ which is some chosen short time after a pulse termination. In the I(t) shown in Figure 7, the t_0_ values are 1, 4, and 8 h, and the t_w_ values are 1, 2, and 3 h.

**Figure 8:**
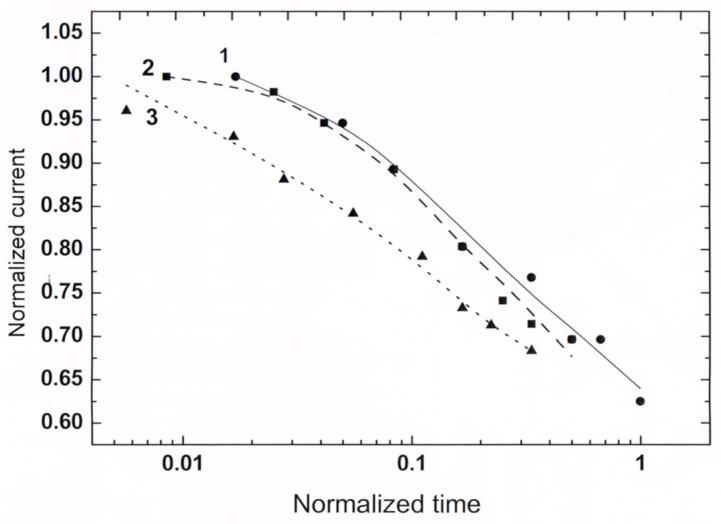
The current decays of Figure 7 as presented by the dependence of the normalized current on the normalized time. This is for the t_w_ waiting–excitation times of one (1), two (2), and three (3) hours. The excitations t_w_s were chosen to enable as many orders of magnitude (t − t_w_)/t_w_ as possible.

The manifestation of the aging behavior is exhibited in Figure 8 by the nearly ideal “collapse” of the normalized current decays. The deviation from ideality in the experiments is known to be due to some “non-glassy” contributions to the conductivity [51,62,71] and, in our case, probably also due to the small contributions of the previous pulses that slightly blur the ideal behavior. 

Having shown that the basic features of a glassy behavior are exhibited by our Si-SiO_2_ nanocomposite, let us examine the possible effect of the QC and its allied CB and CS effects on the I(t) characteristics. As in Section 3, we achieve this by studying the effect of the NCs’ diameter *d* on the I(t) dependencies in the QC regime, which extends here up to the Bohr diameter of *d* = 10 nm. As far as we know, while the glassy behavior of bias excited I(t)s in systems that contain nanoparticles has been studied and confirmed before [33,54,60,61], the effect of *d* on these I(t)s has not been considered. 

**Figure 9:**
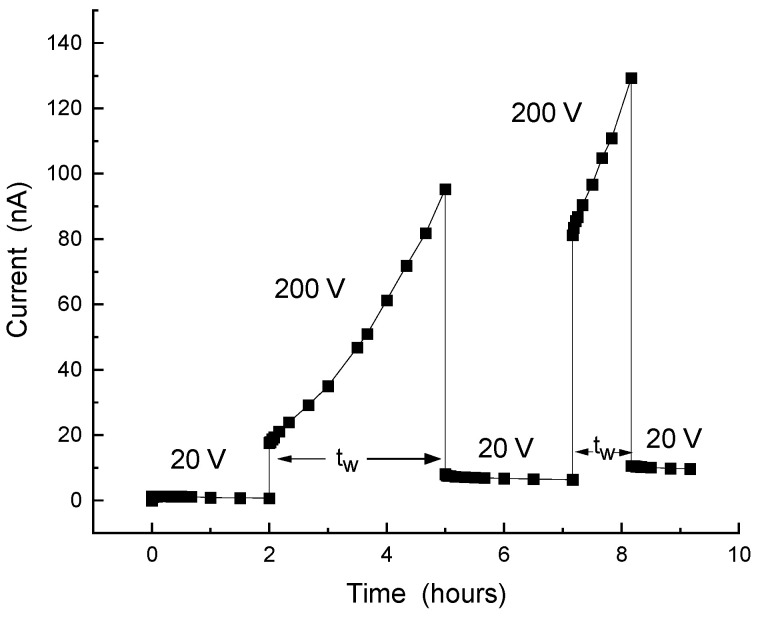
The I(t) dependence of the same *annealed slide sample* for which the results of Figure 7 were obtained, except that the sample here is of x = 47.4 Si vol.% and *d* = 6.7 nm, deeper in the QC regime than that of Figure 7. In particular, note the convex (super linear) rise in I(t) here in comparison with the more common concave rise, as seen in Figure 3, Figure 6, and Figure 7 and in some data of others [60,61].

To examine the possible dependence of the observed I(t) characteristics on the x and *d* values, we repeated the pulse protocol of Figure 7 with a sample of a significantly smaller x and *d*, i.e., closer to x_c_ and deeper in the QC regime. Comparing the I(t) results that are shown in Figure 9 with those in Figure 7, one sees immediately that the I(t) of Figure 9 has a convex behavior rather than the concave behavior that was observed there. Here, we remark that, to our knowledge, no such a superlinear rise in I(t) has been reported for any electronic system for which a glassy behavior has been suggested. We also note here that, and that in general, the excitation part of I(t) has received thus far only little attention in the studies of such systems [53,57]. 

**Figure 10:**
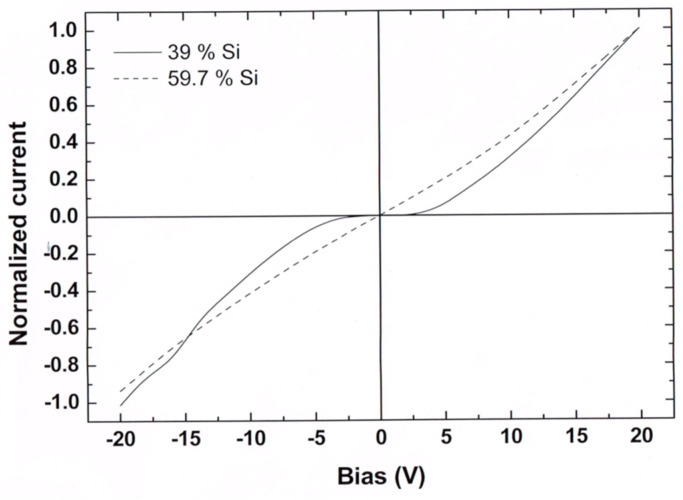
Typical current–voltage characteristics for two nano-Si-SiO_2_ samples that were deposited and annealed together. Their corresponding x values (39 and 59.7 Si vol.%) are associated with *d* = 6.2 nm and *d* = 7.4 nm, respectively.

Turning to explain the *d*-dependent difference in the I(t)s of Figure 7 and Figure 9, we analyze the corresponding behaviors in two steps. First, we consider the dependence of the steady-state I–V characteristics on x and *d*, and then we show how the conclusion derived can be applied for the interpretation of these I(t)s. We start with Figure 10, where we show two typical steady-state current–voltage I–V characteristics that we observed on the same slide sample but for different x (and thus different *d*) samples (see Section 2). One sample (x = 39) is deeper in the QC regime (*d* = 6.2 nm) than the other (x = 59.7 and *d* = 7.2 nm). For the sample of a smaller x, the I–V is superlinear, which indicates a tunnel barrier step, while for the sample of a higher x, the I–V characteristic is nearly linear, which indicates a conduction in a continuous medium. Indeed, for the smaller-x and -*d* samples, one would expect higher (due to the QC effect [82,123]) and wider (see Figure 2) intercrystallite tunneling barriers as well as a higher charging energy [13,87] of the NCs and its corresponding enhanced CB. In contrast, as we noted previously [96], for the higher x and the larger *d*, the conduction is of a diffusive (migration-like) nature, as expected from the Ohmic current through touching NCs. 

Noting the general agreement of the above simple interpretation with previous interpretations of the I–V characteristics [18,19] observed in other nanosystems and in our observations using local electron spectroscopies [11,81], we conclude that in systems of Si NCs embedded in a thin oxide layer [18], the macroscopic I–V characteristics reflect the average sum of the similar local I–Vs. This conclusion also applies to MOS capacitors that make nonvolatile memories [49,50,119,127]. In particular, we note that in these systems it was convincingly demonstrated that the macroscopic I–V characteristics increase in a *convex* (or superlinear) manner that follows the local tunneling spikes, such that each local spike or stair is marked by an observed bias increment [13,19,84,118] which is “due to quantized charging of the NCs and to some electrostatic interactions between the trapped charges and the tunneling current” [49,127]. Here, we attribute the abrupt decrease in the local current spikes to the closing of a corresponding percolation path [128], which is due to an electron capture by a previously uncharged neighboring NC [49,119]. Hence, it is safe to attribute the observed convex I–V dependence to the QC [129] and/or the CB [17,123] effects. It follows then that the Ohmic I–V for the high-x and -*d* samples represents a continuous network of NCs that touch or are connected by a tunneling via a narrow barrier between them [91]. This interpretation of the variation from a linear or a concave I–V behavior to a superlinear or a convex I–V behavior is explicitly supported in [50] where, following the convex macroscopic behavior, it was concluded that the “spherical well-separated NCs with smaller size and lower density” will yield that “the electrical current would be more sensitive to quantized charging of nanoparticles”.

**Figure 11:**
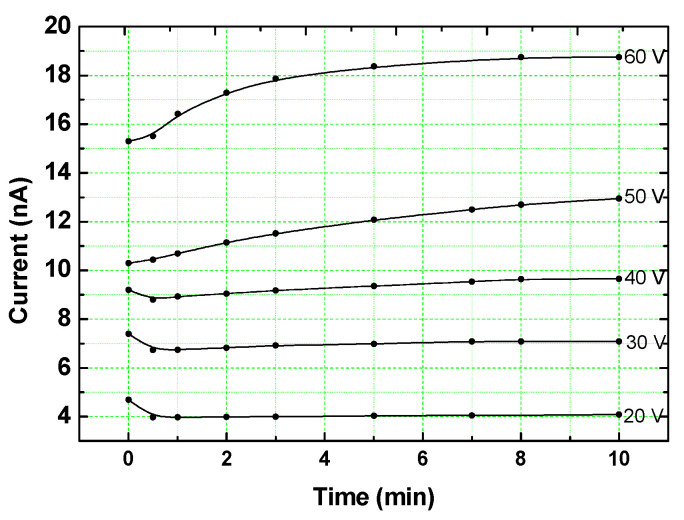
Typical I(t) dependencies in the excitation regime as a function of the excitation bias. The sample used for this measurement is of x = 41.5 Si vol.% where *d* = 6.2 nm. This result was obtained on the same slide sample that yielded the results in Figure 7 and Figure 9. In this figure, as in the following ones, the initial very short experimental overshoot is not shown.

Coming to the second step of our analysis, we recall that in the above paragraph we were concerned with the steady-state I–V characteristics such as in Figure 10 rather than with the out-of-equilibrium transient I(t) characteristics, such as those presented in Figure 7 and Figure 9. In the latter step, we suggest that the *qualitative* similarity between the two types of characteristics can be helpful for the interpretation of the latter characteristics. This approach is justified by the general rule that “any perturbation taking the system out of equilibrium must lead to an increase of the conductivity” [71]. In our case of the excitation mode, the increase in V, F, and t takes the system out of equilibrium, as manifested by their common increase in the value of I. Indeed, the I–Vs in MOSFET devices [49,50,93,119] and in Si quantum wells [18,123] show a behavior that is similar to the observed I(t)s in the drain current in such systems [92] and the behavior that we found in Figure 6. This suggests that one can map the I–V dependence such as in [18] onto the I-t dependence, such as in Figure 6. In order to establish that such a qualitative mapping of the I–V onto the I(t) characteristics applies to the systems and bias excitation that we study here, we examined systematically the V dependence of the I(t)s. This was achieved by using smaller (V < 100 V) bias excitation in comparison with the V = 200 V that we used in Figure 7 and Figure 9. In Figure 11, we show then the variation in the I(t) characteristics with the increase in V as obtained on a sample of a small x and *d* (x = 41.5 Si vol.% and *d* = 6.2 nm). This x and *d* are even deeper in the QC regime and closer to x_c_ = 35 Si vol.% than the sample used for Figure 10. The conspicuous feature in Figure 11 is that the concave excitation characteristics of I(t)s are similar to both those that we found with optical excitation in Figure 3 and Figure 6 here, and they are reminiscent of the concave I(V)s that were found under such an excitation for Si NCs in [18]. 

The other feature to be noted in this figure is that for the very low V values, the initial I(t) decreases with t, while at higher Vs, all the I(t)s turned to rise and to approach a peak. Since the important result for our analysis is the dependence of the I(t)s on V, we leave the discussion of the initial current decline for further below. Concentrating here on the current rise with the increase in V, let us consider the upper two I(t)s of 50 V and 60 V. As seen in the figure, the current rise between these characteristics is of the same order (~5 nA) as is in the current rise during the full time of the excitation of these I(t)s. This establishes that the increase in bias and the increase in time yield similar contributions to the current rise during the excitation, thus justifying our suggestion for a possible mapping of the current dependence on t for a given V onto the current dependence on V for a given t. 

The mentioned mapping within the framework of Equation (1) enables us to explain now the observed I(t)s of Figure 11 in light of the conclusions reached in the literature from studies of the I–V characteristics [18,70,82]. First, for a given t, the increase in V enhances the carriers’ injection, i.e., the increase in *n*, the concentration of mobile carriers, as well as their crossing of the various barriers in the system, as represented by the increase in the mobility μ. On the other hand, for a given V, initially almost all the injected carriers are trapped by some of the NCs so that only very few of them contribute to the conduction. As time goes on, both *n* and the concentration of the charged NCs, which is the concentration of trapped electrons, *n_t_*, increase. However, this increase in *n_t_* opposes the carriers’ injection due to their screening of the applied V, the CB effect, and the carriers’ scattering by repulsion of the charged NCs [64]. This decrease in μ at a high enough value of V and/or a long enough time is manifested eventually by a clear plateau of the observed current. The onset of this plateau marks then the transition from the non-equilibrium state to the selected steady-state conductivity of Equation (1). While this will be discussed later, here we already remark that in systems such as ours, this steady state is not necessarily sustained. In particular, while the concentration of trapped electrons *n_t_* may not vary much, the arrangement of the trapped charges can form a charged landscape that is not favorable for transport, thus reducing the effective μ even further. This can yield to the current decrease with time still during the excitation regime, as also implied by other works [38,49,119] and by the slight decline in I(t) in Figure 6.

Following this basic understanding of the concave I(t)s dependence in Figure 11 and thus of Figure 7, we use the above mapping for the understanding of the I(t)s of Figure 9 by recalling the superlinear I–V characteristics that have been observed in systems of Si NCs [123,129]. As others [119], we [79] have previously suggested, on the basis of the steady-state I–V and C–V characteristics in systems of Si NCs, that in the small-x and -*d* samples, the transport is dominated by tunneling or hopping conduction, while for the larger-x and -*d* samples, it is dominated by migration or simple tunneling between practically touching NCs neighbors [75,130]. Applying then the qualitative resemblance of the I(t) and the I–V behaviors, we conclude that the I(t)s and the I–Vs are affected by the same QC and CB effects. In particular, we suggest that the difference between the concave behavior in Figure 7 and the convex behavior in Figure 9 indicates the stronger manifestation of the local QC-CB effects in the nearly isolated and smaller NCs in the case of Figure 9. This is in contrast with the “Ohmic” behavior of the touching NCs or the narrower barriers in the case of larger-*d* and denser NCs, as in Figure 7.

**Figure 12:**
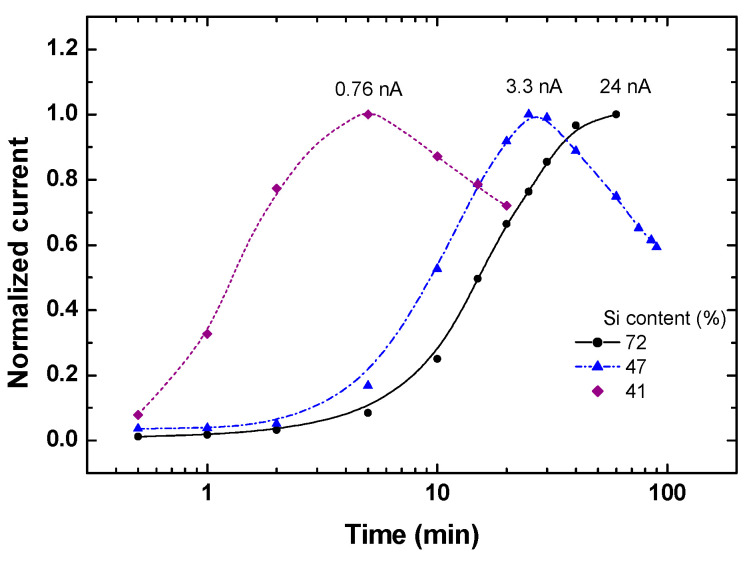
I(t) dependencies of three Si-SiO_2_ samples under the application of a step pulse of 100 V. The samples belong to the same slide sample but differ by their x and *d* values; note that a normalized current value of 1 stands for current values of 0.76 nA for x = 41, 3.3 nA for x = 47, and 24 nA for x = 72. The corresponding *d* values are 6.3, 6.7 and 8.0 nm. The percolation threshold of this slide sample is x_c_ = 35.

Using the above mapping, let us connect our understanding of the role of the *n*/*n_t_* ratio in the observed transients under the influence of the QC and CB effects on the three features of the glassy behavior. In our nanosystems, the only property that accounts for the memory and the aging is the stored electrostatic charges, i.e., the value of *n_t_*. Then, the sluggishness of the I(t)s relates to the hopping conduction within the percolation network [131], as set by the landscape of the *n_t_*s. Since *n_t_* and the *n*/*n_t_* ratio are determined by the depth of the local trapping well [18], they vary with the energy variations associated with the QC and CB effects. In particular, this suggests that the I(t) transients associated with glassy behavior will vary with the variation in the principal parameter that controls these effects, i.e., the value of *d*. To test this expectation experimentally, we used a slide sample that was annealed at 1200C so that the entire silicon phase consisted only of NCs (see [16] and Figure 2). Indeed, this slide sample exhibited a nice quantum confinement effect of intensive photoluminescence (PL) in the x range of 12–32 Si vol.% [75], which indicates that at least for x < 32 Si vol.%, the Si NCs are well isolated in the SiO_2_ matrix [9,86]. Correspondingly, we studied the excitation characteristics under a bias of 100 V, which is intermediate between that of Figure 11 and that of Figure 7 and Figure 9. The I(t) characteristics that we obtained for three values of *d* (indicated in the figure by the corresponding x values) are shown in Figure 12.

The conspicuous feature of Figure 12 is the peak (i.e., there is a decrease in the I(t)s at larger times), although the entire measurement is in the excitation regime. This is consistent with the photocurrent decrease during the illumination excitation regime that we found for our Si-SiO_2_ samples in Figure 6, and as found in [85], where the variation in *d* is clearly accounted for by the QC shift of the optical absorption edge. While the increase in the peak current values with x > x_c_ here is expected, the striking and new realization here is that the time evolution is sensitive to the value of *d* and following [85] explicitly to the QC and/or CB effects. That the observed peaked behavior is not a spurious effect or an effect that is special to our particular Si-SiO_2_ composites is evidenced by the observation of such a peak during the excitation of high-resistance samples, in a system of (non-spherical) metal islands [61]. This establishes that the NCs in our Si-SiO_2_ composites play the same role as the metal islands there. The present conclusion is of major importance in the context of the present work since the glassy behavior has been well confirmed in [61], which strongly supports our above suggestion that the composites studied here exhibit a glassy-like behavior. This peak has not been explained in [61], and as far as we know, no interpretation of this peak has been given in any other work. Also, no *d* dependencies, such as in Figure 12, have been previously reported. 

Being already familiar with the rise in I(t) and the current plateau in Figure 11, the main basic question that arises is the reason for the decay of I(t) past the plateau while the system is still within the excitation regime. To answer this question, we start from the consensus regarding the decay relaxation of I(t) that follows the termination of the excitation in an electrical system with a glassy behavior [23]. This is that the approach to equilibrium is accompanied by the spatial reorganization [70], randomization [28], or repartition [38] of the localized charges, which follows the corresponding minimization of the system’s energy. In electrical systems as here, the relevant energy is the electrostatic energy of the landscape of the charged NCs [13], and the relevant transport is the hopping of the charging electrons between a charged and an uncharged nanoparticle [15]. Indeed, it has been proposed for the post-excitation regime that the rearrangement of the charging electrons yields also the “minimization of the conductance” upon the approach to equilibrium [38], but no specifics were given to that. Here, we suggest that this effect applies also during the excitation, since then also the repartition of the corresponding charged NCs with time jeopardizes the preceding percolation network of touching NCs, which eventually brings the decrease in I(t). This interpretation is consistent with the advancement in the observed peak with the decrease in *d*, as in Figure 12, since the decrease in *d* enhances the QC and CB effects in the electrostatic landscape. As our interpretation involves only charged and uncharged nanoparticles, it accounts for both our results in Figure 12 and the results obtained for the high-resistance samples of metal islands in [61]. Of course, we note that the results in [61] and in Figure 12 were obtained in the excitation regime, while the above considerations, as in [28,38,70], were concerned with the current decay after the termination of the excitation. However (see also below), following the realization that the increase in time always tends to drive the system towards equilibrium, we consider that in the excitation regime this process takes place in parallel to the tendency towards steady state. Correspondingly, we suggest that while in Figure 11 we saw the time regime of the domination of the approach towards the steady state, in Figure 12 and in [61], we also see the eventual dominance of the tendency towards equilibrium. 

**Figure 13:**
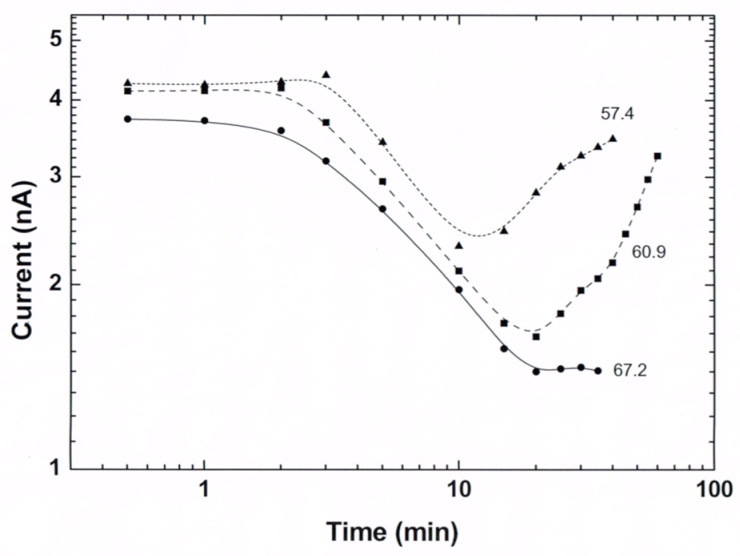
I(t) excitation characteristics due to a step pulse of a small V = 20 V bias. This is as in Figure (4.5) but here for three low-resistance samples of higher x (57.4, 60.9 and 67.2 Si vol.%) and larger *d* (7.2, 7.4 and 7.6 nm) values.

The authors of [61] have also reported the excitation I(t) in their low-resistance samples, for which they found the opposite behavior to that of the peaked-like I(t) dependence in Figure 12, i.e., an initial decline and then the rise in I(t). Now, we recall that we found a hint of such an inverted peak behavior in our high-resistance (small-x and -*d*) samples but under the very small bias values in Figure 11. To validate that this behavior of the low-resistance samples in [61] is also found in our low-resistance samples, we measured the behavior of I(t) in our own low-resistance samples (i.e., in higher-x and -*d* samples). In the measurement, we used a low (V = 20 V) bias in order to avoid the elimination of the inverted peak effect of high (V > 50 V) bias that can be seen in Figure 11. Indeed, the I(t) dependence that we show in Figure 13 exhibits the same inverted peak behavior that was found in [61] for the low-resistance ensembles of metal islands. We further note that the initial decline in I(t) was also observed, and for the same time scale as in the present work, in nano-CdSe [82]. The common peaked and inverted peak behavior in Figure 12 and Figure 13 here and in [61] shows that in spite of the differences between the two systems (semiconductor and metallic), the same property controls the conductivity in both types of systems. This property is obviously the nanosize of the particles in them, and the apparent common mechanism is the CB effect. Again, the importance of this in the context of the present work is that the observed behavior here is the same as in [61], thus providing strong support to our suggestion that the systems studied here exhibit a glassy behavior. 

The above similarities also indicate that the common general transport mechanism in the above-mentioned systems is the variation in the *n*/*n_t_* ratio that we described following Figure 11. In particular, our interpretation of the results in Figure 13 is that under a small bias, the initially available large *n* charges the NCs towards saturation, thus yielding a high *n_t_* that opposes the injection and the transport of the latter electrons. Then, with a further injection of electrons with time, the *n*/*n_t_* ratio increases, and the conductivity is dominated by n. This interpretation is consistent with our mapping of t onto V (as seen for V ≥ 40 V in Figure 11), since the increase in V increases the injection and overcomes the barriers to electron propagation in the system. Hence, the difference between the peaked I(t) in Figure 12 and the inverted peak in Figure 13 can be explained now. In Figure 12, the conduction is determined by hopping under the Coulomb blockade conditions, while in Figure 12, once *n* “overcomes” *n_t_*, the conduction in the high-x samples takes place via the percolation network that consists of touching NCs. 

**Figure 14:**
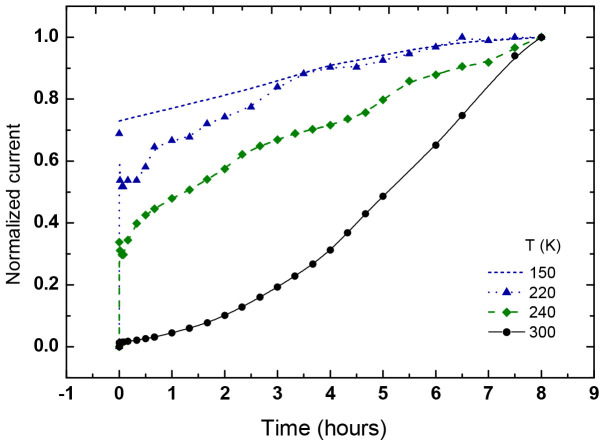
I(t) characteristics in the excitation regime under four ambient temperatures, as obtained for the sample that was used for Figure 9 but under a long step-like pulse bias of 200 V. The value of x was 47 Si vol.%, and the value of *d* was 6.7 nm. The reference for the normalization of the current in this figure is the arbitrary value of I(t = 8 h). Note that, as in Figure 3, the original non-normalized I(t) increases with temperature. The dashed line at t = 0 indicates where the initial very short experimental overshoot occurs. As seen in Figure 11 and Figure 13, I(t) has an inverted peak in the first few minutes, which is eliminated under the combined effect of a high V and a high T. The important observation, however, is that the decrease in temperature has the same effect on the curvature of the I(t)s as the effect of the increase in x and *d* from the one shown in Figure 9 to the one shown in Figure 7.

As we have already seen in the case of CdSe (Figure 3), the temperature dependence can provide a useful tool for the interpretation of the observed I(t)s in nanosystems in general and due to QC-CB effects in particular. We mentioned already in Section 3 that this tool has been used thus far, almost exclusively, for the study of the temperature dependence of the I(t)s in the decay relaxation regime of [48,58,66]. Here, however, we found it useful to study the temperature dependence of the I(t)s in the excitation regime. The results of the corresponding measurements under the high bias of 200 V for temperatures in the 150 and 300 K range are shown in Figure 14.

This is based on the sample for which we obtained the results in Figure 9. To emphasize the change in the I(t) features with the variation in temperature, we present the currents by their normalized I(t)/I(t = 8 h) values, but one should note that the currents are temperature-activated, increasing from 150 to 300 K. These results show that under the application of a high V to samples of small x and *d*, the decrease in temperature yields a change from the convex I(t) dependence at room temperature (as in Figure 9) to the concave slow excitation (as in Figure 7). As with the increase in bias in Figure 11, the increase in temperature here contributes to the enhancement in the non-equilibrium state during the excitation. We also see here that for the same V and T, there is a slowdown in the current increase with time. This suggests that, as in the decay relaxation regime, there is also here an effect of the rearrangement of the landscape of the charged NCs towards the equilibrium. The observed behavior can be interpreted microscopically as follows. For the present small-x and -*d* sample, a higher temperature enables the hopping conduction to cross the bottlenecks in the NCs system and thus to select the shortest paths for the percolation. When the temperature is reduced, the only available paths are the few very tortuous ones that consist of touching NCs where no thermal activation of the conduction is required. Except for the low-temperature contribution of the touching NCs that we suggest here, the present interpretation is quite similar to the one proposed in [38] for systems of granular metals. There, it was suggested that the rearrangement of the charges is accompanied by the increase in the average distance that needs to be traveled by the hops in order to maintain the current. Following this, we suggest that the reduction in the conductivity with the decrease in temperature, as shown in Figure 14, is due to both the macroscopic (“glassy”) effect of the arrangement of the charged NCs and the microscopic effect of the reduction in the activated hopping [16,22,122] between the Si NCs. To connect this suggestion with the QC and CB (i.e., the *d*-dependent) effect, we recall that by comparing Figure 7 and Figure 9 we found the same variation in I(t) from convex to concave with the increase in x and *d*. As there, we explain the conductivity of the high-x and -*d* sample by the conduction through the percolation network of touching semiconductor NCs [22,84,86] or touching metallic grains [15,130]. This latter part of the network is still available at low temperature and low bias, as was demonstrated by the concave behavior for small bias in Figure 11. On the other hand, for the small-x and -*d* sample, there is a need for a higher T and/or V in order to “compete” with the percolation network of touching NCs by activating current bottlenecks in the rest of the system. This activation is manifested by the (non-linear) I–V convex dependencies that are known to accompany tunneling and hopping conductions. The forgoing self-consistency of the T and *d* dependencies shows that the observed I(t)s are a combination of two, a priori separable contributions, the particular transport mechanism and the glassy dynamics of the landscape of the charged particles in the system. Moreover, the similarity between our results in Figure 12 and Figure 13 and the results on nanometallic systems [61], for which the glassy behavior was convincingly confirmed, provides further support to the glass-hopping–percolation model that we suggest here. 

**Figure 15:**
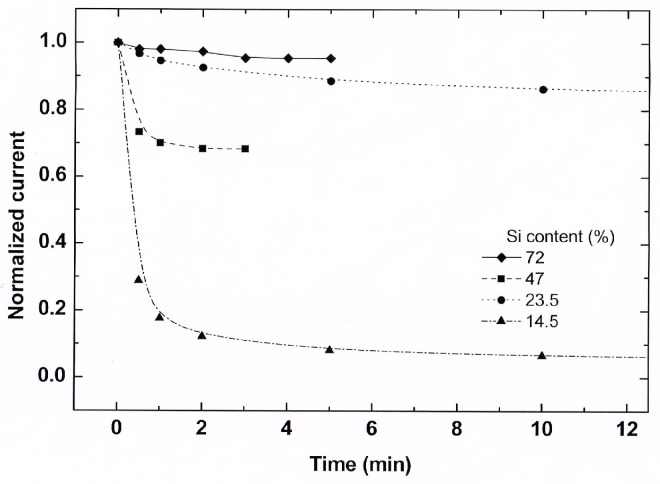
Normalized I(t)/I(t = 0) decay dependencies measured under the common background bias of V = 10 V, after the termination of the excitation bias of V = 100 V. This is for four x and *d* samples from the same slide sample that was annealed at 1200C. Here, the x values were 14.5, 23.5, 47, and 72 Si vol.% with the corresponding *d* values of 3.9, 4.9, 6.7, and 8.0 nm. Note that here both the values of I(t) and I(t = 0) increase with the increase in x.

Finally, having accounted for the I(t) features during the excitation regime, as in Figure 12 and Figure 14, we recall that most of the corresponding previous studies of the I(t)s [28,42,60,61,119] were concerned mainly with the current decay regime. To show, however, that the conclusions derived following Figure 14 regarding the transport mechanism also apply to the latter regime, we studied the x and *d* dependence of the I(t)s on a slide sample that was annealed at 1200 C, where, as seen Figure 2, the entire Si phase consists only of NCs. In the measurements, we followed the decay relaxation characteristics after the termination of a 100 V bias excitation, where the termination took place past the peak, as in Figure 12. In Figure 15, we present the results with the normalized [I(t) − I(t = 0)]/I(t = 0) current, where I(t) is the measured current due to a post-excitation background bias of 10 V, and t = 0 is the chosen time after which the current decay was followed. The results for the four samples used that are given in the figure show that the time constant of the decay increases by at least a factor of 10 between that of a sample of a very small x and *d* and that of a sample of a large x and *d*. Turning to the interpretation of the observed time constants, we start with the sample of x = 14.5, which is far below the percolation threshold (of x_c_ ≈ 35), finding a very fast decay compared to all the decays that we have seen above. This is well explained as due to the simple dielectric (displacement current [112]) response of the small-x composite, where the NCs (as seen in Figure 2) form a very dilute electrically disconnected system. Upon approaching x_c_ from below by x = 23.5, we see that with time the system relaxes, as to be expected for hopping processes [36,113], which in the present case enable the conduction between clusters of touching NCs. Then, for x = 47, which is above x_c_, the relative contribution of this hopping to the conductivity is reduced, and for x = 72, the conductivity is fully determined by the touching NCs, thus becoming very weakly dependent on t. This interpretation of the results in Figure 15 in terms of a percolation–hopping network is indeed in accordance with our conclusion following the results in Figure 14, i.e., that with the decrease in T, the electrical conduction changes from being dominated by this network to being dominated by the network of touching NCs. The role of hopping here and in the ensembles of CdSe NCs that we described in Section 3 will be further discussed in the next section. 

## 5. Summary and General Discussion

In this section, we summarize and discuss the main particular and general advancements made in our work regarding the glassy-like behavior of semiconductor nanosystems in which the QC and the related CB and CS effects have been well established by many [1,5,6,18,21,39,73,76,112,122,123] and by us [3,9,75,77]. We consider these advancements in three consequent parts. In the first, we establish that indeed these glassy features of the conductivity transients are exhibited by the CdSe and Si-SiO_2_ semiconductor nanomaterials that we studied, and we mention the common and different transport mechanisms in these nanomaterials and the viscous glass. In the experiments, we followed the changes in the electrical current transients I(t) upon the variation in *d,* the average diameter of the NCs. As far as we know, no *d* dependence of observed I(t)s has been previously reported for any semiconductor nanosystem. In the second part, following the complexity of the combination of the glassy mechanism and carrier transport mechanisms, we suggest a new approach for analyzing the observed transient currents for all the electrical systems that exhibit a glassy-like behavior and we utilize it for the interpretation of the temperature dependence of these transients in general and in the nanosystems that we study here in particular. In the third part, we reveal the source of the glassy behavior in the electrical properties of both metallic and semiconductor nanosystems. In particular, we argue that the glassy features that we observed have a deep reason rather than being an accidental, empirical, or generic description of experimental observations and that this reason is the similarity between the statistical–structural landscape of the classical glass [27,29,31,35,37] and that of the nanosystem. As far as we know, this is the first explanation for the emergence of the electrical glassy behavior in systems composed of nanoparticles. 

Starting from the glassy features that we observed, we recall in particular that in both our nano-CdSe and nano-Si-SiO_2_ systems the time constant of the decay relaxation past the excitation τ increases with the increase in *d.* For the Si-SiO_2_ nanocomposites, we also found that, as shown in Figure 7 and Figure 9, under the application of high bias for a long time, the I(t) characteristic in the excitation regime changes its shape from concave to convex upon the decrease in *d*, while for intermediate bias and short times, we observed a peaked I(t) (see Figure 12), where the peak shifts to longer ts with the increase in *d*. Then, we noted that the peaked and the inverted peak features of I(t) that we found in Figure 12 and Figure 13 for the excitation regime are very similar to those observed using the same two-probe lateral configuration in the study of metallic nanosystems [61]. This suggests that even though there are significant differences between our semiconductor Si-SiO_2_ nanocomposite and the nanometallic system, the mentioned similarity can only be attributed to the CB effect, which is common to both systems. The particular importance of this conclusion is that the distinct feature of the glassy behavior, the aging, has been convincingly confirmed proven in [61], thus strongly supporting our evidence in Figure 8 that our Si-SiO_2_ nanocomposite also exhibits the aging property that is the main characteristic of a glassy behavior. We have to remark, however, that some similar I(t) features, though usually with shorter time constants, have been observed in various photoconducting systems, and these were interpreted to be due to recombination processes by particular defects [44,94,101]. We suggest, however, that this is not the case in the present work. First, the recombination mechanism in defects that are not associated with the QC effect should not change significantly over the narrow *d* range that we studied. In contrast, such a change in *d* around the Bohr diameter in the nano-QC regime is likely to yield changes in the electronic structure and transport [1,7,10,79] that, as observed here, affect the transients in nanosystems in a significant way. Then, the similarity of the I(t) dependence here to that in granular metals [66,132,133] and discontinuous metal films [51,61] strongly indicates that the I(t)s that we observed are a result of the nanosize of the particles and not even due to possible defects in an oxide layer that is in contact with them [132]. In fact, the latter argument is important for MOSFET [22,63,119] and quantum well structures [47,58,99], where the transient behavior is well known to be associated with the embedded NCs in them. Hence, we conclude that in the systems studied here, as in all the nanosystems mentioned above, the CB and CS mechanisms [1,2,18,19] strongly affect the transient transport.

Considering the implication of the above for the relation between the transport mechanism and the glassy behavior, we start by noting that in the systems that we study, the charge carriers travel between the *stationary* NCs, and in their travel, they propagate in a percolation network that provides the lowest possible macroscopic resistance [128]. In the systems of present interest, this will be in paths that consist of the smallest number of possible QC [134] or CB [16] barriers as well as by the lowest possible repulsive electron scatterings from the already-charged NCs. It is expected then that the smaller the NCs, the larger the resistance of the sample will be, as is indeed the result that is shown in Figure 12. This *d* dependence is consistent with the established evidence that the electron transport mechanisms in our nanosystems can vary from those of electrons between touching [86] or tunneling [15,18,22] to hopping [135] and to variable-range hopping [21,131,136,137] between non-touching nanoparticles. Hence, there is variation from the diffusive–ohmic conductance for the touching of large NCs (as in Figure 7 for the larger *d*) to that of bias-activated conduction (as in Figure 9 for the smaller *d*). To connect the above transport mechanisms with the glassy behavior, we recall that in the classical “viscose” glass, the system consists of disordered (atomic or larger) particle ensembles, where few of them may be thermally excited to wander due to the global-like collective vibrations of their neighbors [24,25,26,27,31,37]. The macroscopic manifestation of this is the slower decrease in the viscosity in the glass with the decrease in temperature. Correspondingly, if one considers the nanoparticles to be charged, the system is expected to yield similar collective glass-like global effects of the electrical conductivity. However, in the systems of interest here, the wandering particles are the electrons rather than the nanoparticles that are stationary. It is clear then that the interesting phenomena associated with the electrons’ conductivity, as here, will take place at lower temperatures than those of the viscosity of the glass. On the other hand, it is apparent that in systems that exhibit a glassy-like conductivity, the observed I(t) will be determined by the complex combination of the global arena of charged NCs and the electron transport mechanisms that were mentioned above. In particular, one notes that two different time scales may or may not be involved in the observed I(t)s. 

Turning to the second part of this section, we encounter the above-mentioned difficulty in the understanding of the observed I(t)s that follows the complexity of the CGB by beginning with the separation of the two possible time variations that contribute to the measured I(t)s rather than by using the less-transparent holistic practice that has been suggested theoretically [33,61,71]. As shown below, this will enable us to understand various, so far unexplained, features of the I(t) characteristics here and in other works. Our approach to the mentioned difficulty is based on the understanding that two different physical principles govern the transient conductivity of a system on its way to equilibrium. The first principle, hereafter FP, is that during this transition, the medium in which the transport takes place favors the lowest possible energy state [29,138], and this energy is minimized at equilibrium [28]. In the electrical nanosystems considered here, this energy is the global electrostatic energy of the landscape of the charged nanoparticles [13,132]. The smaller the crystallites and the more isolated they are from each other, the deeper the energy of this landscape and the longer the time it takes for the electron discharging (or electron detrapping) from a charged NC [17,18,118]. The manifestation of this energy minimization can be represented by assigning an effective temperature to the excited non-equilibrium state T*, which is higher than T, the ambient temperature of the system both during and after the excitation [54,74]. Then, the tendency towards equilibrium (i.e., the cooling from T* towards T) is accompanied by the time-dependent “repartition of the charges between the grains which minimizes the energy (and the conductance) of the system” [38]. Unlike the above electrostatic energy, the electrical conductivity is determined by the different basic principle of the minimal energy dissipation [139] in a system. This second principle, hereafter SP, is that the electrical current favors the particular percolation network of minimal macroscopic resistance out of the many possible networks that are a priori available in a system [140]. It is important to note that the generality of this principle is beyond the classical statistical–geometrical model of the percolation where all the resistors have the same value, since the existence of some high-resistance elements in the backbone of the percolation cluster modifies the current network with respect to that of the basic percolation model, yielding a corresponding lower conductivity of the system [128]. In our nanosystems, these high resistors are mainly associated with the local environments of the charged NCs. 

Now, it is important to note that the above FP and SP are not compatible with each other since they relate to two different physical principles. In particular, while the first involves the global landscape of the charged NCs in the system, the latter is determined by the connectivity of a selected part of it [16,132]. It is clear then that the observed resultant transient current is determined by the time evolution of the percolation network that changes with the changing landscape of the charged NCs. This is for both upon the approach towards the steady state during the excitation and the approach towards the equilibrium, after the termination of the excitation. We suggest that the above non-compatibility between the consequences of the FP and the SP is the reason for having thus far only qualitative descriptions for the conductivity transients in the nanosystems. These include nonspecific statements such as “the transport in the granular metal film is a highly selective process” [19] or that the “Coulomb correlations play a major role in the transport” [70] within films of CdSe NCs. 

It appears to us then that in order to interpret the measured I(t)s, one has to start with the evaluation of the separate possible contributions of the FP and the SP. Starting from the FP, we expect to find very long (stretched exponential [32], logarithmic [33,51,55] or power law [29]) decays and an aging effect. However, one has to note that such behaviors can arise from different systems’ scenarios. The recognition of this seems to be important when one considers the controversy regarding the “electron glass” behavior in granular metals [66,67,69] as follows. In nanosystems such as ours and metallic nanoparticles, the electrostatic energy is that of the landscape of stationary charged particles, while in the electron glass [57], the energy of the system is all due to that of the “mobile” electrons that interact only between themselves. This accounts then for the observation that in nanoparticle systems [60,61] the glassy behavior is observed at much higher temperatures than in the electron glass. 

Turning to the SP, we recall that the basic possible interparticle transport mechanisms of the electrons in systems of nanoparticles are diffusive migration between touching particles [86] and interparticle tunneling [96], interparticle hopping [135,141], and variable-range hopping [131] between non-touching particles. However, since the SP is manifested by the resultant percolation [140], this heterogeneity complicates the interpretation of the observed conductivity in these nanosystems. For example, in the nanosystems studied here, there is a competition between the transport via touching NCs and the temperature- and/or bias-activated hopping-like transport that shortens the backbone of the touching NCs. Describing the complications associated with the FP and the SP themselves let us also consider the further complication that follows their interaction, which is the one that is actually observed in the macroscopic transient measurements. In particular, since the rearrangement of the charged particles tends to form a homogenous spatial distribution, some charged NCs can break the paths of uncharged touching particles, thus enhancing the hopping contribution to the conductivity. On the other hand, the corresponding FP lowering of T* also suppresses possible hopping channels. All this is in accordance with the basic general rule that the approach to the equilibrium state will always yield a decrease in the conductivity (and vice versa [71]). On the other hand, this further illustrates that the finding of a glassy behavior in our systems of NCs and in other systems of nanoparticles [60,61] is a clear indication that the decay of the transient currents is not a simple result of the transport [15,141], i.e., of the SP, but is associated with changes in the transient conductivity due to both the FP and the SP and their interaction. 

Having this conclusion, let us show now how starting with a separate consideration of the FP and SP effects can be useful for the interpretation of I(t) dependencies that were reported in the literature in general and for the temperature dependencies of the decay time constant τ(T) that we reported following Figure 3 and Figure 14 in particular. First, we recall that when the system is out of equilibrium its extra (here electrostatic) energy, i.e., T*, is larger than the ambient temperature T. Thus, when the concentration of charged NCs hardly changes, the energy of the system decreases with time, mainly due to the rearrangement of the charged landscape [70,142]. A priori, this is the reason for the very slow current decay with time, rather than the particular transport mechanism (such as the sluggish hopping) that we associate here with the SP. In other words, if the FP would have been the only effect that determines the I(t)s, the decrease in T would reduce the value of T* and thus lengthen τ, the time constant of the rearrangement [38]. In particular, the participation of the SP effect due to the charge carriers’ transport between the NCs can have a similar or a different τ(T) dependence. For example, upon the rearrangement of the charges in the system, the tunneling conduction can also become thermally activated in order to overcome the widening of the average distance between the charged particles [38] but with a shorter decay constant than that of the rearrangement. On the other hand, for low temperatures, the conduction may be controlled by the available subnetwork of uncharged touching particles so that τ(T) will still be dominated by the rearrangement. A similar or different behavior from that of τ can be expected for photoconductors [58,143] where (unlike Equation (5)) the recombination time can increase with the increase in T for shallow traps or exhibit the opposite behavior for deep traps [114]. Hence, while in many electronic systems the resultant observed decay relaxation time τ_m_ is extended with decreasing temperature [43,66,73,101], the expected activated behavior of τ, as in Equation (5), is not universal. In fact, while our observed τ_m_ on the Si nanocomposite (as seen in Figure 14) is consistent with the activated τ, it is not consistent with the opposite behavior of τ_m_ that we found (see Section 3) in our nano-CdSe. To explain the latter behavior, we mention the observed peak in the measured τ_m_(T) dependence in some quantum well systems where τ_m_(T) was found to saturate [48] or decrease [58,59,84,100,102] along some T ranges below that peak. Hence, the deviation from the temperature-activated behavior must be associated with the effect of the transport mechanism, so the observed τ_m_ represents not only the combined FP and SP effects but also their interaction. In other words, the mentioned decrease in τ_m_ with decreasing temperature does not necessarily mean the domination of the SP but rather a combined τ_m_ that is just lower than the activated τ value that would have been obtained [66] without the involvement of the SP effect. Following this, we interpret the τ_m_(T) dependence in our photoconducting nano-CdSe, for which we have previously found a hopping-like behavior [77], to be a reflection of the interacting FP and SP effects. This indicates that while the glassy behavior still dominates and the aging effect is maintained, the observed decrease in τ_m_(T) with decreasing T just represents the influence of the hopping relaxation with a shorter decay time [113] over the temperature range that we studied. Similarly, this explains the peaked τ_m_(T) in [58] as a reduction in τ_m_(T) with respect to the expected τ(T) of the FP-only effect but without changing the dominating activated nature of the resultant τ_m_(T). On the other hand, the temperature-activated τ_m_(T) behavior, as in Equation (5), which we found for the nano-Si-SiO_2_ composites is simply explained by noting that in these systems the conduction for x > x_c_ is dominated by the percolation network of touching or near-tunneling conduction between the NCs (see Figure 2) so that the temperature dependence of the SP effect is minor, yielding that the τ_m_(T) is practically dominated by the temperature-activated glassy effect of the FP. The above two cases demonstrate that the detailed understanding of the experimentally observed I(t)s in electrical systems that exhibit a glassy behavior can benefit by starting with the analysis of each of its FP and SP components, noting that these I(t)s are combined resultants of both of them. As far as we know, our present approach to analyze the glassy-like behavior of the electrical conductivity and the utilization of it, such as for the understanding of temperature dependence of the corresponding transients, has not been suggested previously. 

Finally, we turn to evaluate whether there are deep physical reasons, rather than just an empirical–experimental resemblance, between the features of the electrical transient found here and those known for the classical mechanical glass. For this evaluation, let us start by recalling the basic property of glass, which is still a great challenge to explain [26,27]. This is that during a mild cooling of a liquid-like glass towards its amorphous (solid-like, noncrystalline) state, there are relatively very few changes in the positions of the particles (say, atoms or molecules), while the viscosity increases over many orders of magnitude, and practically, the system never reaches the equilibrium crystalline value [25]. This behavior is referred to as the slow dynamics of the glassy state. To explain the mentioned “discrepancy” between the spatial structure and the global dynamics, it was proposed that the initial stage of the corresponding slowdown is determined by thermally activated jumps of a single particle out of its surrounding neighbors [27,35]. A simplified explanation of the involved dynamics is that upon the approach to equilibrium in general, and with the lowering of the ambient temperature in particular, the energy that is required for the “delocalization” of a particle can be accumulated only by thermal fluctuations that result from collective thermal excitations over a scale that is larger than that of its nearest neighbors. The particle’s environment within this scale, which is known as the cage [24,35], expands upon the approach to equilibrium. Correspondingly, the increase in the cage with the decrease in the ambient temperature involves more particle hops in order for it to get out of the cage. Considering the duration of a hop, “the coupling between cage size and duration controls the average behavior” [35].

Having this basic concept of the cage, let us turn to a simplified phenomenological analogy between the nanosystems studied here and the above-described cage in the glass. In both systems, there are wandering particles that are thermally detrapped. In the glass, these are atoms or molecules, and in the nanosystems studied here, these are the electrons. In analogy with the confinement of the particles in the cage of the glass, in the nanosystem, the electrons are confined to their environment by the CB. Considering then that the electron can overcome this confinement when the thermal energy that it accumulates is larger than the capacitive-confining energy of the CB [13], we can define an effective CB cage by the spatial scale of this confinement. Correspondingly, the motion of the electron within this “QC and CB cage” is a hopping process [113,134,135] that is not too different from that of the above-described particle hopping in the viscous glass [31,36]. This analogy accounts for the common slow dynamics as well as for similar temperature dependencies of the corresponding transients. In fact, this explains why the processes involved in our Coulomb glass of nanoparticles take place at much lower temperatures than those involved in the mechanical properties of a glass. In the glass, the particles that wander are atoms or molecules, while in our nanosystems, the moving particles are the electrons (rather than the NCs on which the electrons are trapped). This realization also explains the higher temperatures involved in the transients of the nanosystems, where the electrons are confined to the CB cages, in comparison with the temperatures expected and found in the electron glasses which involve only interactions between Anderson-localized electrons [57]. Hence, considering the temperature range in which the I(t) relaxations have been measured in this work, we suggest that the nanosystems that we studied in this work can be characterized as an intermediate glass system between that of the atomic arrangement in the mechanical glass [31,35] and the electron-only collective relaxation in the electron glass [57]. This justifies our original labelling of the CdSe and Si nanosystems studied in this work as Coulomb glasses in order to distinguish them from the widely studied [52,57,68,71] electron glasses. 

## 6. Conclusions

The work presented here on ensembles of semiconductor nanocrystallites (NCs) was concerned with their transient conductivity during both the excitation from and the relaxation towards the equilibrium state. In particular, in this study, we have measured the dependencies of I(t) and the corresponding transient current characteristics on the size of the NCs in such ensembles of CdSe and Si NCs for which we have previously shown the presence of quantum confinement (QC) and Coulomb blockade (CB) effects. Our present results show that the glassy-like slow decay of I(t) is sensitive to this size, thus revealing the influence of the QC and CB effects on the glassy features that we found. The justification for our interpretation of the results in terms of a glassy behavior was strongly supported by the comparison of our observed I(t)s with those obtained in different systems of nano-semiconductors and nanometallic particles in which the glassy behavior was well established. This led us to the more general conclusion that the main feature that determines the glassy behavior in all these systems is the nano-nature of the NCs rather than defect-related recombination effects, as in bulk semiconductors. Correspondingly, we have further suggested that the deep reason for the glassy behavior in these nanosystems is that the CB in them plays the same role as that of the cage in the classical glass system and its related viscosity. By the variation of the applied bias and ambient temperature, we have also shown that upon the system’s approach to equilibrium the observed I(t)s also reflect the percolation route selection of the electron current under the variation in the landscape of the charged NCs. Following our I(t) analyses in this work, we conclude that the observed I(t)s in electrical systems that exhibit a glassy behavior in general, and in nanosystems in particular, should be interpreted in light of two very different basic physical principles: the minimization of energy that determines the glassy behavior and the minimization of energy dissipation that determines the electrical transport. We thus propose that for the evaluation of the mechanisms that determine an observed I(t) one should first appreciate the role of each of these principles and then consider the observed I(t) as a superposition and/or an interaction of their corresponding effects. We have shown that using this approach provides an understanding of thus far unexplained features of the observed I(t)s in general and for nano-semiconductor and photoconductor systems, such as quantum dot and quantum well devices, in particular.

## Data Availability

The data presented in this study are available on request from the corresponding author.
